# Management of post-chemotherapy residual mass in patients with metastatic nonseminomatous germ cell tumors of the testis

**DOI:** 10.4103/0970-1591.60448

**Published:** 2010

**Authors:** John P. Fitzgerald, Barbara Ercole, Dipen J. Parekh

**Affiliations:** Department of Urology, University of Texas Health Science Center at San Antonio, Texas, USA

**Keywords:** Cancer, testis

## Abstract

The basis of treatment for advanced germ cell tumors is chemotherapy and surgical resection of residual disease. Surgery has maintained its role in staging and therapeutic management. Despite these advances, much of the outcomes depend on proper patient selection. Complete removal of all post-chemotherapy residual masses remains the standard of care in the treatment of advanced nonseminomatous germ cell tumors both within and outside of the retroperitoneum.

## INTRODUCTION

Germ cell tumors account for 90-95% of all testicular tumors, broadly divided into seminomatous and non-seminomatous germ cell tumors (NSGCT). As a class, they are the most commonly occurring cancers in men between the ages of 15-35 years, representing approximately 1% of all malignancies seen in men.[1] Advances in combination chemotherapy along with improvements in surgical technique have revolutionized the treatment of metastatic germ cell tumors. The development and introduction of cisplatin-based chemotherapy and a nerve sparing approach to the retroperitoneal lymph node dissection have translated into superior disease specific survival, while decreasing morbidity when compared to the treatment in the past. Testicular cancers have become one of the most curable of the solid neoplasms at the present time. The focus of this article is on the surgical management of patients with non-seminomatous germ cell tumors after receiving chemotherapy.

## RATIONALE FOR SURGERY

Optimal management of residual mass following chemotherapy for NSGCT is a subject of ongoing debate. In general pure necrotic tissue is found in 50%, teatime in 35% and viable germ cell in 15% of the post-chemotherapy resected masses.[2] It is due to the diverse nature of the subsequent histology that surgical excision of the residual mass usually becomes necessary as this dictates further management of the patient.

In addition to the diagnostic benefit served by surgical excision of the residual mass, a therapeutic component is served. This is clear when the resulting histology is viable germ cell tumor and teratoma. In a study from Indiana University,[3] 10% of patients who received primary chemotherapy and 90% of patients who received salvage chemotherapy were found to have viable germ cell tumor in resection specimens. Incomplete resection was found to have a higher rate of cancer specific mortality with 80% in those partially resected and 40% with complete resections. Teratomas found in the retroperitoneum are not responsive to chemotherapy as well as radiation therapy. Moreover, they have the potential of local growth with invasion into critical surrounding structures as well as malignant transformation to different histological subtypes.

Benefits served by the excision of the residual mass include a definitive histology, accurate pathologic staging and future management directives. Complete removal of residual masses grants therapeutic control over the retroperitonium. These benefits are delivered with acceptable morbidity and mortality rates.

## IDENTIFICATION OF THE RESIDUAL MASS

Among patients with good-risk disease as per the International Germ Cell Cancer Collaborative Group risk stratification (IGCCC), 70% will have no detectable lesions in the post-chemotherapy setting. The remaining 30% of patients will have a persistent finding on imaging.[4] In the absence of rising tumor markers, surgical excision is the standard of care. The size criteria of a significant residual mass on CT scan vary widely from institution to institution. A value of < 20 mm is considered normal by some, while a value of 15 and 10 mm is the standard elsewhere.[5] In one study, a 35% false negative rate was found to exist when the cut off was set to 20 mm, a value accepted as normal in many institutions.[6] This has lead some to routinely perform RPLNDs on all patients who had post-chemotheraputic residual masses. Size of the residual mass is quite often a poor predictor of subsequent histology. The group from Memorial Sloan Kettering Cancer Center observed a significant likelihood of viable germ cell tumor or teratoma if the residual mass was greater than 10% of the initial tumor size or if the prechemotherapy diameter of enlarged lymph nodes was 30 mm or more, despite any reduction in size after treatment.[7]

## POST-CHEMOTHERAPY RETROPERITONEAL LYMPH NODE DISSECTION CLASSIFICATION

The Indiana Classification[8] of post-chemotherapy RPLND divides these patients into standard or complicated. Patients with disseminated testis cancer after receiving a regimen of cisplatin based chemotherapy and normalized tumor markers with residual disease in the retroperitoneum, chest, mediastinum or neck are classified as standard post- chemotherapy RPLND. The remaining RPLNDs are grouped as ‘complicated’. The complicated group consists of four subgroups. The first is salvage, which are those men who are status post second line salvage and have normalized tumor markers. Desperation RPLND denotes those men who have had second line salvage chemotherapy and have persistent tumor markers, indicating chemoresistant cancer persists. Redo RPLND are those men that have a previous RPLND with infield recurrence. The remaining small group is the unresectable RPLND, who have a grim prognosis.

## PATIENT SELECTION

**Figure 1:**
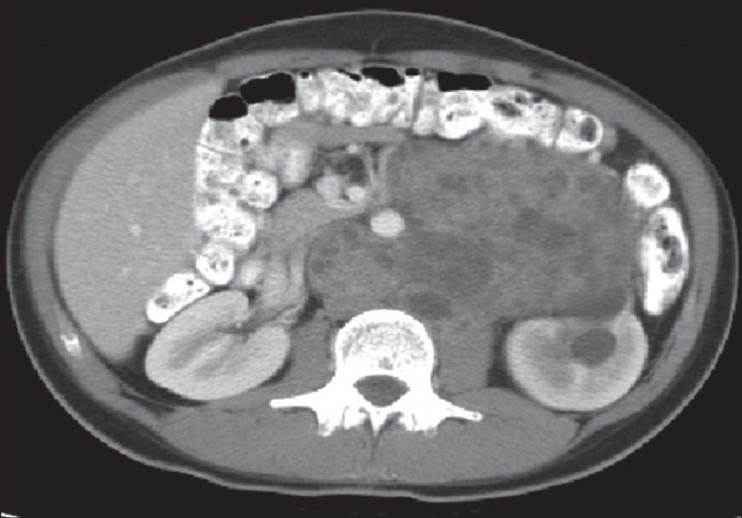
CT scan of patient with normal tumor markers, after chemotherapy

Indications for surgery after primary chemotherapy are varied and depend on a number of factors. The histology of the primary tumor and the finding of a residual mass on imaging in large part dictate the post chemotherapy management. Primary tumor histology can predict the behavior of the post-chemotherapy mass. When teratoma is seen in the primary resection there is an association with teratoma being found in the residual mass. In one study,[2] 82% of patients with teratoma in the primary tumor, normalized post-chemotherapy markers with a residual mass were found to have teratoma. The finding of a residual mass on CT scan is also cause for controversy as a normal CT scan may fail to reliably exclude the presence of residual tumor or teratoma in the uncontrolled retroperitonium. In a study by Fossa,[9] 13 of 37 patients with normal post-chemotherapy CT scans, demonstrating no lymph nodes > 10 mm and normal tumor markers had teratoma or viable tumor in the resected tissue [Figure 1]. It has been estimated that the false negative rate for CT scans after first line chemotherapy is roughly 20-25%.[6] This highlights our present inability to accurately identify a subgroup of patients with normal post-chemotherapy CT scans and tumor markers in whom adjunctive surgery can be safely omitted. With size being an inconsistent predictor of histology, any detectable mass mandates removal. This broadly includes those men who have undergone primary chemotherapy with rising tumor markers who are given second line high dose chemotherapy with bone marrow support with subsequent normalization of tumor markers. Men with increasing tumor markers, despite second line combination chemotherapy, should be considered for desperation RPLND. Patients whose primary tumor contained no teratoma component and whose tumor markers have normalized with lymph node size decreasing more than 90%, although still at risk for relapse, can be managed with active surveillance and imaging.

## POST-CHEMOTHERAPY RPLND

**Figure 2:**
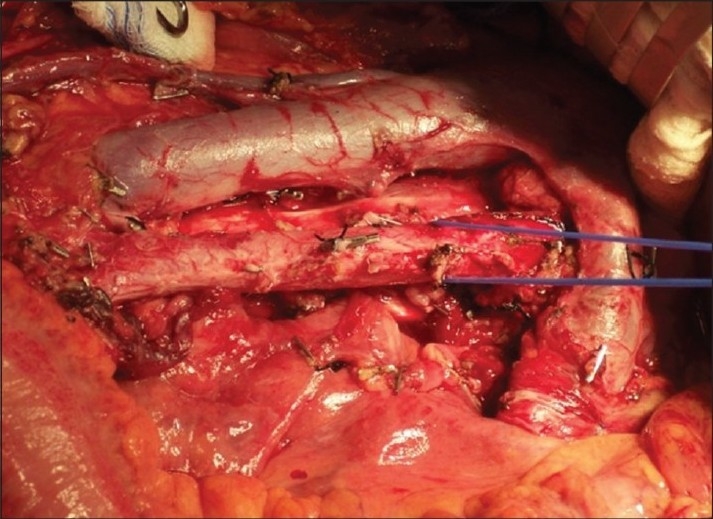
Same patient as in Figure 1 after excision of the residual mass and complete bilateral RPLND

Before embarking on surgery, a complete metastatic work up should be performed. A CT scan of the chest, abdomen and pelvis should be obtained about six to eight weeks after the last cycle of chemotherapy. Tumor markers should be current as well as pulmonary function parameters. Although CT scan suffices in most patients [Figure 2], MRI is the preferred imaging modality in patients with suspected vascular invasion. Once the mass is detected, the timing of the RPLND should be prompt and the resection complete. Hendry *et al*,[10] demonstrated a significant benefit (83% vs. 62%) in progression free survival and in cancer specific survival (89% vs. 56%) in those men undergoing immediate surgery. In a contemporary study,[11] it was shown that half of the men with local recurrence after post chemotherapy RPLND had been incompletely resected at the time of the primary surgery.

The extent of surgery has undergone much debate and modification over the past 20 years. Historically, RPLNDs in this setting have included a complete bilateral suprahilar dissection spanning from ureter to ureter, including the crus of the diaphragm, extending to the bifurcation of the common iliac arteries. The role of limited post chemotherapy RPLND has been explored in depth. Rabbani *et al*,[12] reported on 50 testicular cancer patients who underwent post chemotherapy RPLND as a full bilateral dissection, a modified-template dissection with resection of the residual masses or excision of the mass alone. Those patients who received a bilateral dissection (38 of 39) did not develop any tumors outside the boundaries of a modified template. All nine patients who underwent a modified template dissection with resection of the residual mass were free of relapse in the 55-month follow up period. Of the two patients who underwent resection of the residual mass alone, one experienced two recurrences attributed to incomplete resection.

Aprikian *et al*.[13] examined the utility of intraoperative frozen section analysis to dictate the surgical procedure. If the mass demonstrated necrosis, a modified template RPLND was performed. If teratoma or viable tumor was found, then a bilateral approach was used. 21(53%) of patients showed necrosis in the frozen section analysis with 18 (85%) confirmed by permanent sections. Three (14%) of these patients had a recurrence with none in the retroperitoneum. The other 18 had no recurrence in the follow up period. This study also suggests that a limited RPLND maybe safe in patients with necrosis in the frozen section.

More recently, the work of the Witthuhn[14] examined the outcomes of 74 patients undergoing modified unilateral (n=38) or full bilateral PLND (n=36). There were no recurrences within the dissection field, but two patients experienced outlying recurrences. Of note, three patients in the study died, two from progressive disease and one from a surgical complication. 84% of those who underwent the modified dissection retained antegrade ejaculation.

Ehrlich *et al*,[15] examined 50 patients with advanced germ cell tumors to identify a subset of patients that might be appropriate for modified template resection. Their analysis suggests that low volume disease, with a left sided primary may benefit from only a modified template. They also recognized that right-sided primaries demonstrate a 20% crossover rate, and may not benefit from a limited dissection. Retroperitoneal surgery for testis cancer has been evolving over the past 30 years due, in part, to better understanding of neuroanatomy and germ cell tumor biology. Despite this, the extent of post-chemotherapy RPLND still remains controversial.

Recent studies highlight limitations on the oncologic efficacy of the limited post chemotherapy RPLND. Fossa *et al*,[16] reported an incidence of 5.7% relapse rate outside the boundaries of a modified template in patients subject to the limited dissection. Similarly, Carver *et al*.[17] reported their experience on incidence of disease extending outside the boundaries of five modified template RPLND in current use. They noted in 532 patients a 7-32% incidence of extratemplate recurrence, depending on which template was used. They report a recurrence rate of 4% to 32% for right-sided templates but also an 11% to 32% for left sided templates. This study provides important insight into the importance of the extent of RPLND for patients with retroperitoneal disease after chemotherapy. In addition the study also points out that tumor which recur after chemotherapy are chemoresistant, which casts some doubt in the efficacy of further non-surgical management. Although the understanding of the nodal landing sites has improved, when considering bulky post-chemotherapy disease subject to a limited dissection, the reported success rates of limited template dissections may be flawed and premature since long term follow up in these patients is lacking.

Despite the support in the literature that bilateral dissection may not be required in every case, strong consideration should be given to proper patient selection. The modified post chemotherapy RPLND is reported as a safe option in men if the lesion is well defined, less than 5 cm and is in concordance with the primary landing site of the primary testicular tumor.[11] Those men with high volume post chemotherapy, residual diseases are best served with a full bilateral dissection.

## MORBIDITY OF RPLND AFTER CHEMOTHERAPY

The post-chemotherapy RPLND is a formidable surgery due to complexity of the operation and the desmoplastic changes that occur after chemotherapy. The incidence of complications increases significantly when compared to a primary RPLND. The most common complications include wound infections, paralytic illeus, transient hyperamylasemia and atelectasis. In two per cent of patients, more serious complications can be seen, including acute renal failure, chylous ascites and obstructive illeus.[2]

## POST-CHEMOTHERAPY EXTRA-RETROPERITONEAL RESIDUAL MASSES

Post-chemotherapy, approximately 35% of patients will have radiographic evidence of extra-retroperitoneal masses. These sites may include brain, liver, bone, mediastinum and lymph nodes. The findings of Shayegan *et al*,[18] demonstrated a histological difference between retroperitoneal histology and extra-retroperitoneal histology, meaning necrosis is not always the rule when considering other sites. Carver *et al*.[19] report that 50% of patients with extra-retroperitoneal residual masses will harbor teratoma or viable germ cell tumor at these sites. Complete surgical resections of all residual extra-retroperitoneal masses, therefore, are indicated.[20]
